# Transcranial direct current stimulation for balance and gait in repetitive mild traumatic brain injury in rats

**DOI:** 10.1186/s12868-021-00633-4

**Published:** 2021-04-17

**Authors:** Gahee Park, Jee Hyun Suh, Soo Jeong Han

**Affiliations:** 1 Department of Rehabilitation Medicine, College of Medicine, Ewha Womans University, 1071 An-Yang-Cheon Ro, Yang-Cheon Gu, Seoul, 07985 Republic of Korea; 2 Department of Rehabilitation Medicine, Bundang Jesaeng General Hospital, 20, Seohyeon-ro 180 beon-gil, Bundang-gu, Seongnam-si, Gyeonggi-do 13590 Republic of Korea

**Keywords:** TDCS, Mild traumatic brain injury, Balance, Motor evoked potential

## Abstract

**Background:**

Balance impairment and lack of postural orientation are serious problems in patients with repetitive mild traumatic brain injury (mTBI).

**Objective:**

To investigate whether anodal transcranial direct current stimulation (tDCS) over the primary motor cortex (M1) can improve balance control and gait in repetitive mTBI rat models.

**Methods:**

In this prospective animal study, 65 repetitive mTBI rats were randomly assigned to two groups: the tDCS group and the control group. To create repetitive mTBI model rats, we induced mTBI in the rats for 3 consecutive days. The tDCS group received one session of anodal tDCS over the M1 area 24 h after the third induced mTBI, while the control group did not receive tDCS treatment. Motor-evoked potential (MEP), foot-fault test, and rotarod test were evaluated before mTBI, before tDCS and after tDCS. The Mann–Whitney U test and Wilcoxon signed rank test were used to assess the effects of variables between the two groups.

**Results:**

Anodal tDCS over the M1 area significantly improved the amplitude of MEP in the tDCS group (p = 0.041). In addition, rotarod duration was significantly increased in the tDCS group (p = 0.001). The foot-fault ratio was slightly lower in the tDCS group, however, this was not statistically significant.

**Conclusion:**

Anodal tDCS at the M1 area could significantly improve the amplitude of MEP and balance function in a repetitive mTBI rat model. We expect that anodal tDCS would have the potential to improve balance in patients with repetitive mTBI.

## Introduction

Mild traumatic brain injury (mTBI), also known as concussion, is a head injury that temporarily affects brain function. They commonly occur in adolescents and young adults who practice sports and driving. Previous studies have shown that 144,000 children and adolescents visit emergency departments for mTBI per year, [1] with full incidence of mTBI estimated to be as high as 3.8 million annually in both adolescents and adults [2]. The symptoms of mTBI include non-motor symptoms such as headache, loss of consciousness, and memory loss, but there are also motor symptoms such as balance impairment, lack of motor coordination and decreased dynamic motor function [3, 4]. Because of these symptoms, patients with mTBI have difficulty returning to exercise or daily life. In addition, when mTBI occurs repetitively, prognosis is poor. Parkinson's disease and chronic traumatic encephalopathy, which both affect balance and motor coordination, may occur after repetitive mTBI [5]. Repetitive mTBI is a source of significant economic burden in terms of days lost from work and costs related to medical treatment [4]. Recently, with the increasing practice of exercise and driving increase, the frequency of repetitive mTBI has also increased. However, there are few studies on the treatment of sequelae caused by repetitive mTBI. Furthermore, previous studies have focused primarily on non-motor defects rather than on motor deficits. It is important to further evaluate these motor deficits caused by repetitive mTBI as they may be a cause of long-term problems for patients.

Transcranial direct current stimulation (tDCS) is a form of neuromodulation that uses a constant, low direct current delivered via electrodes to the head. tDCS is a non-invasive, easy to handle, and low-cost technique that induces regional changes in neuronal excitability [6]. Previous studies have reported that tDCS has a therapeutic effect in patients with neurological disorders, such as Parkinson’s disease, Alzheimer’s disease, and stroke [7–9]. tDCS has emerged as a promising therapeutic tool to improve balance and postural control [6]. In a previous study, tDCS applied at the primary motor cortex (M1) in individuals with cerebral palsy and healthy young adults resulted in improved balance [6]. However, no study has assessed the effect of anodal tDCS on balance and postural orientation in patients with repetitive mTBI.

The brains of humans and rats are anatomically similar, which makes rats good models for studying human brain disease [10]. In humans, mTBI varies with rotational force, acceleration-deceleration, and degree of impact [11, 12]. Therefore, it is difficult to fit the baseline similarly. Therefore, in this study, we want to verify the effectiveness of anodal tDCS at the M1 area by making rat model to check the baseline function and making a uniform rat model with repetitive mTBI. The purpose of this study was to investigate the changes in electrophysiology, balance control, and postural orientation as an effect of anodal tDCS at the M1 area in rat models with repetitive mTBI. We hypothesized that anodal tDCS at the M1 area would improve balance impairment after repetitive mTBI.

## Material and methods

This prospective, randomized animal study was approved by the Institutional Animal Care and Use Committee of Ewha medical research institute (approval number 13–0235). Sixty-five healthy male Sprague–Dawley rats (Orient Bio, Seongnam, Korea) weighing 220–280 g supplied by a single-source breeder were used in this study. Based on a previous literature, six-week-old rats were used in this study as this age corresponds to the late juvenile to early adulthood stage in rats, which is the common age of mTBI occurrence in humans [13]. Hormonal levels influence the cortical excitability and neurotransmitter levels which affect the tDCS response [14–16]. And the male would receive more current at the cortex than female due to the cortical bone structure [17]. Furthermore, males make up larger percentage of cases than females in mTBI [18]. For these reasons, in this study, only male rats were included. The animals were housed in a rodent facility at 23.0 ± 1.0 ºC with 12-h light–dark cycle, and had free access to tap water and regular rat chow. All animals received human care in compliance with the National Institutes of Health guidelines for the use of experimental animals. This study was carried out in compliance with the ARRIVE guidelines.

### Experimental design

**Fig. 1:**
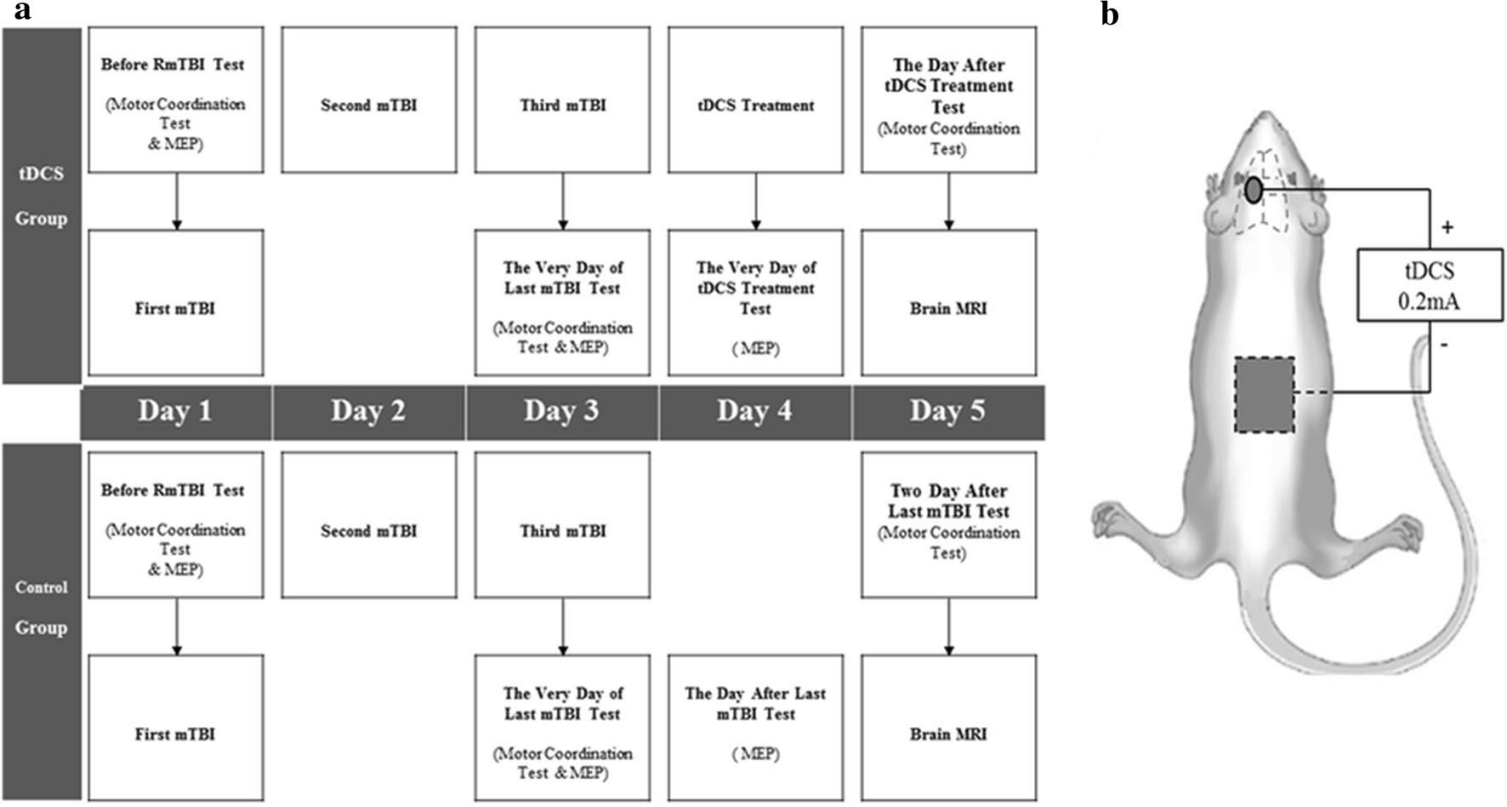
Experimental arrangement and the position of electrodes during tDCS. **a** Experimental arrangement. Before induction of repetitive mTBI (day 1), MEP and behavioral tests are performed on the rat model. After pre-mTBI tests, the first mTBI is induced in the rat models. On days 2 and 3, second and third mTBI is induced in the rats to establish the repetitive mTBI rat models. After the third mTBI, MEP, and motor coordination studies are done (day 3). On day 4, anodal tDCS at M1 area is applied to the tDCS group. On day 4, 1 h after tDCS, MEP is done. On day 5, behavioral tests and brain MRI are done. **b** The position of anodal and cathodal electrodes during anodal tDCS. Circle means anodal electrode and square means cathodal electrode. Cup-shaped anodal electrode was attached to the skin over the left M1 area, and rectangular rubber cathodal electrode was positioned on the trunk

The treatment time and test schedule are shown in Fig. 1a. Each of the 65 rats used were randomized to the tDCS (n = 33) and control groups (n = 32). The tDCS group received one session of anodal tDCS treatment at 24 h after repetitive mTBI (day 4), while the control group did not receive tDCS treatment after repetitive mTBI.

### Animal preparation

All procedures and evaluations, except motor coordination studies, were performed under anesthesia. Anesthesia was initiated with an intramuscular injection of tiletamine/zolazepam (15 mg/kg; Zoletil®, Vibac, France).

**Fig. 2:**
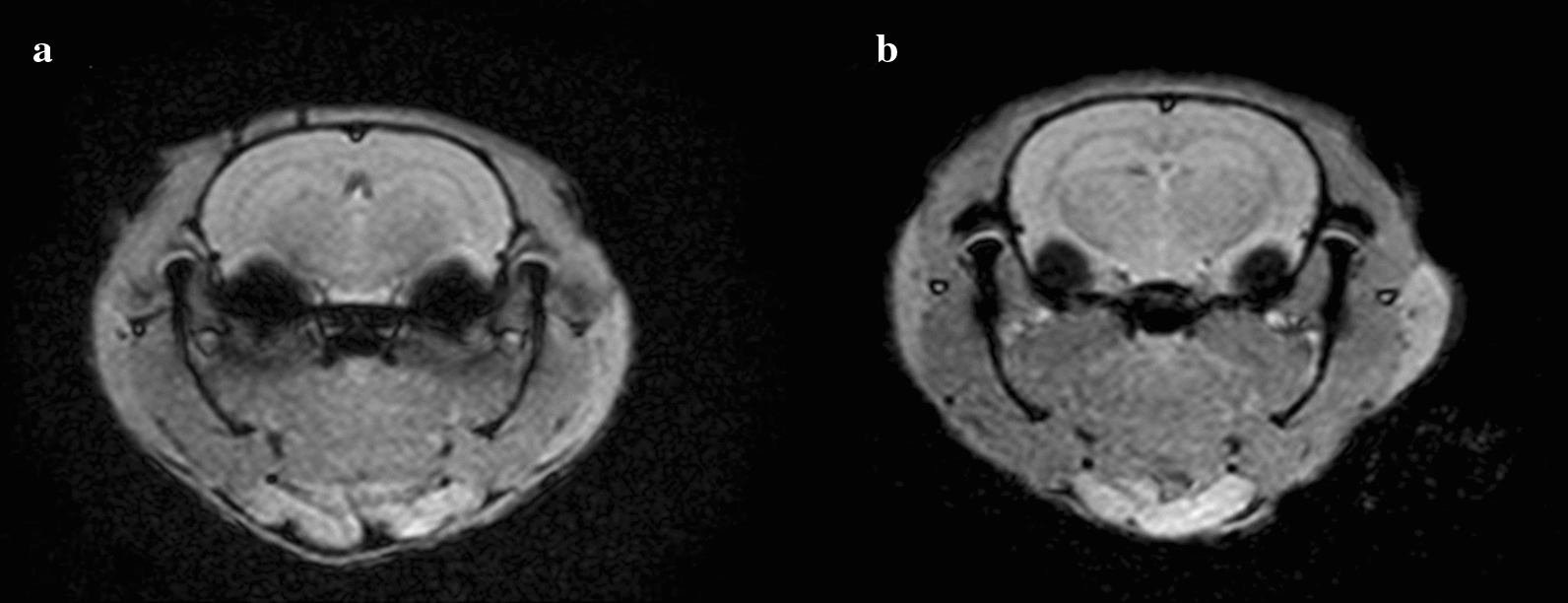
Brain magnetic resonance imaging (MRI) of repetitive mTBI rats. **a** Brain MRI of control group, **b** Brain MRI of tDCS group. Brain MRI showed no significant pathological features after repetitive mTBI in either the control or tDCS groups

**Fig. 3:**
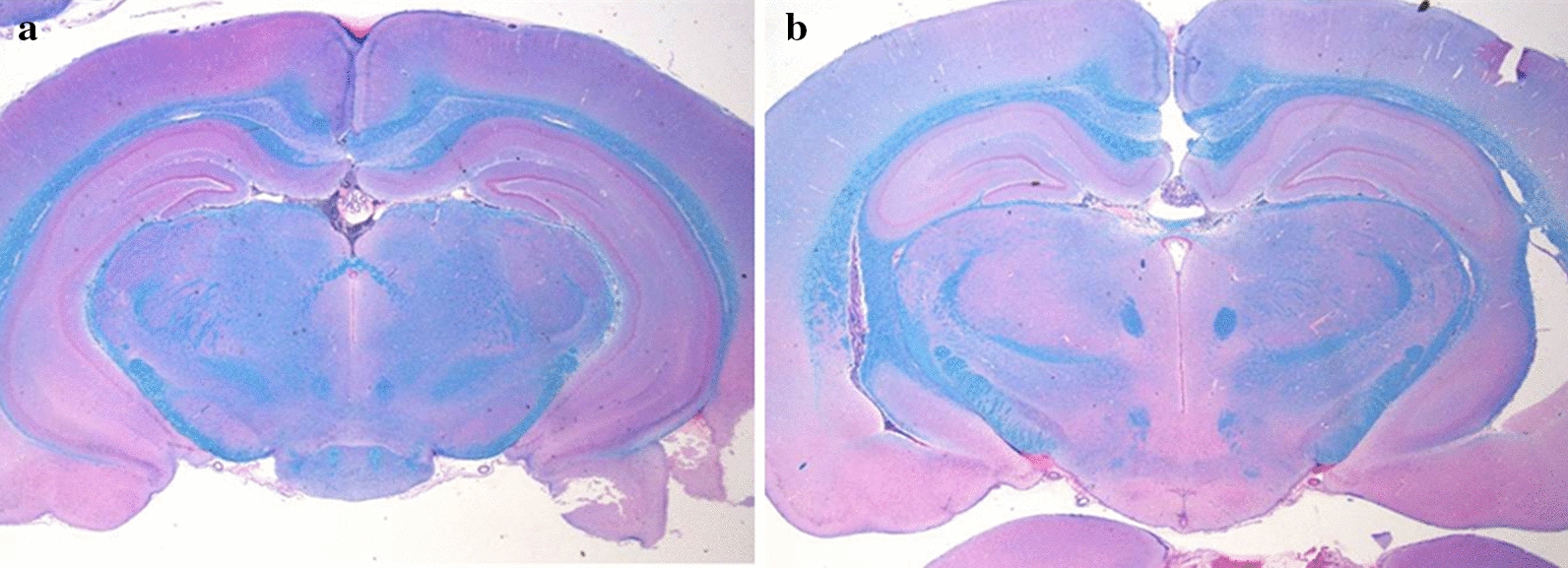
Hematoxylin and eosin staining in repetitive mTBI rats. **a** Brain MRI of control group, **b** Brain MRI of tDCS group. Histochemical analysis showed no morphological changes in either the control or tDCS groups

The induction of repetitive mTBI was conducted for 3 consecutive days (days 1–3) in all rats (Fig. 1). mTBI was induced in rats using a modified weight-drop device and a protocol previously described by Tang et al. [19]. A 175 g steel weight was briefly dropped from a height of 30 cm through a polyvinyl chloride tube with an inner diameter of 11 mm terminating on the bregma of the rat. The rat was placed on a wooden plate and fixed with Velcro in the prone position. To evaluate the possibility of a skull fracture or brain hemorrhage, we conducted brain MRI study on all rats after completing the study on day 5. The rats were anesthetized with Zoletil® and placed in a 5 cm inner diameter, 4-element phased-array, animal-dedicated surface coil (Chenguang Medical Technology, Co., Ltd, Shanghai, China). The strength of the MRI magnet was 3 T. The MRI protocol used was a T2-weighted image sequence (repetition time/echo time = 650/22). The slice thickness was 3.0 mm and the matrix scan size was 512 × 512 pixels. Brain MRI showed no significant pathological features, such as skull fracture, brain hemorrhage, or diffuse axonal injury after repetitive mTBI (Fig. 2a, b). The histological examination was performed to rule out the presence of brain damage resulting from repetitive mTBI at day 5. Brains of rats were removed and fixed in 10% neutral buffered formalin after being euthanized in a closed chamber with 10% carbon dioxide. Coronal brain Sects. 15 µm thick were cut using a microtome, mounted on glass slides, and stained with hematoxylin and eosin. Light microscopy was used for evaluating morphological changes in brain tissue at 100 × magnification. Histochemical analysis showed no visible morphological changes in either the control or tDCS groups (Fig. 3a, b).

### Experimental procedures

After three inductions of mTBI, the rats in the tDCS group received a single session of anodal tDCS under anesthesia, 24 h after the last induction of mTBI (day 4, Fig. 1a), while those in the control group received only anesthesia without anodal tDCS stimulation. The fur around the bregma was removed to ensure tight attachment of the anodal electrode. An 10 mm diameter (0.785cm^2^ contact area), cup-shaped anodal electrode was attached to the skin over the left M1 area, 3 mm to the left and 2 mm in front of the interaural line (Fig. 1b) [20]. The 30 × 30 mm^2^ rectangular rubber cathodal electrode was positioned on the trunk and wrapped with a bandage (Fig. 1b) [21, 22]. The salt-free, chloride free electrically conductive gel was filled in cup-shaped anodal electrode and applied to the rubber cathodal electrode. A constant direct current was applied via stimulator (PhoresorII®, IOMED, Salt Lake City, UT, USA), with an intensity of 0.2 mA and a current density of 0.255 mA/cm^2^ for 30 min [21, 23, 24]. tDCS was performed by a single experienced physiatrist.

### Measurements

**Fig. 4:**
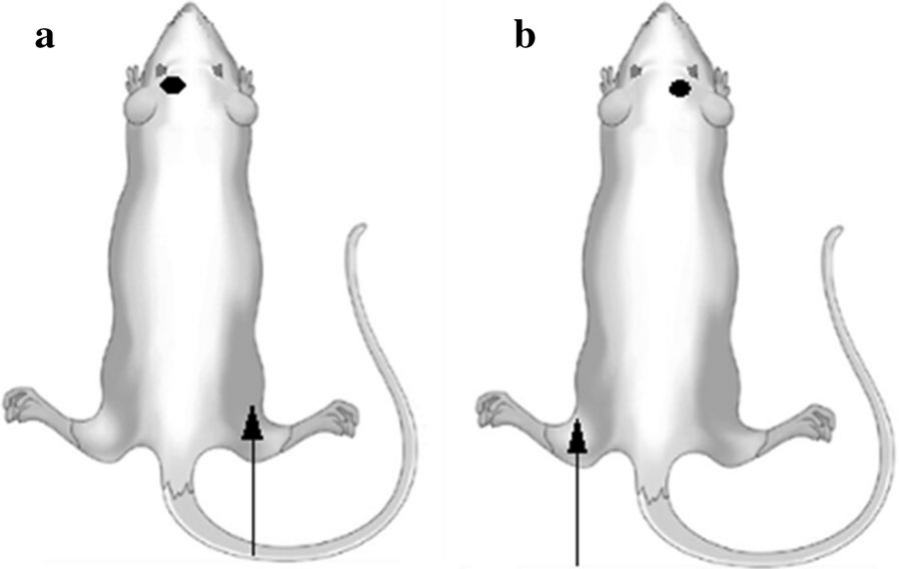
Schematic representation of the MEP study. **b** Right MEP is recorded from the tibialis anterior muscle of the right hindlimb, which results from stimulation of the left motor cortex. **b** Left MEP is recorded from the tibialis anterior muscle of left hindlimb, which results from stimulation of the right motor cortex. The active needle electrode used for the MEP study is indicated by the white arrow, and the black circle indicates the stimulation site of transcranial magnetic stimulation

To evaluate the functional integrity of the motor system, transcranial motor-evoked potentials (MEPs) were evaluated. MEPs are muscle action potentials elicited by transcranial magnetic brain stimulation [25]. In this study, MEP measurements were evaluated pre-mTBI (day 1), immediately post-mTBI (day 3), and 1 h post-tDCS (day 4) to evaluate the excitability of the corticospinal pathway. MEPs at the bilateral tibialis anterior muscles of the hind limbs were evaluated. The right MEP was recorded from the tibialis anterior muscle of the right hindlimb, which resulted from left motor cortex stimulation (Fig. 4a). The left MEP, which was used as the control variable, was recorded from the tibialis anterior muscle of the left hindlimb, which resulted from the right motor cortex stimulation (Fig. 4b). The active needle electrode was inserted into the belly of the tibialis anterior muscle, and the reference needle electrode was inserted into the distal part of the tibialis anterior muscle. The ground electrode was placed on an opposite footpad. MEPs were recorded using a Medtronic Keypoint® (Medtronic Inc., Jacksonville, FL, USA) at a sweep velocity of 5 ms with a sensitivity of 200 µV. The band-pass filter was set at 20–10 kHz. Single-pulse transcranial magnetic stimulation was administered using a magnetic stimulator Magstim® (Magstim Company, Whiteland, Wales, UK) and a figure-eight magnetic coil (diameter per loop = 50 mm, peak magnetic field = 4.0 T). The center of the coil was positioned on the motor cortex, whose center was anterior and lateral to the bregma on the contralateral side of the hindlimb, where the active needle electrode was inserted (Fig. 4a,b). A total of 20 MEPs were recorded at 10 s inter-stimulus intervals [26]. TMS intensity was recorded as percent machine output(MO), with 100% corresponding to the maximal amplitude electrical current conducted through the magnetic coil. We set the stimulation intensity to 100% MO [24]. The intensity of the stimulation was maintained constant throughout the procedure. The average latency of three representative waves was calculated with reference to previous research, [26] and the highest value was analyzed for peak-to-peak amplitude in the mean of three waves; latency was defined as the interval before the initial deflection.

To evaluate balance control and postural orientation, the foot-fault test [27] and the rotarod test were conducted. The foot-fault test has been found to objectively demonstrate impairments in motor coordination and sensorimotor function, and rehabilitation effects after ischemia in rodents [28]. The rotarod test was used to assess motor coordination and balance alterations in rodents [28]. These tests were conducted pre-mTBI (day 1), on the day of the last mTBI (day 3), and 2 days post-mTBI (day 5, 1 day post-tDCS), to eliminate anesthetic effects. In the foot-fault test, an elevated 52 × 40 cm^2^ stainless steel metal grid with grid cells of 3 × 3 cm^2^ was used. The rats were placed in the center of a metal grid and observed for one min via video recording. A foot fault was considered when the hind limbs fell between the grid cells or when the paw was correctly placed on the grid but slipped during weight bearing for hind limbs.^29^ The foot-fault ratio was obtained by dividing the number of foot faults by the total number of footsteps on hind limbs [29]. A rotarod treadmill was used to conduct the rotarod test. The rotation speed was set at 15 rpm, and a rat was placed at the center of a 9 cm diameter metal roller. The trial lasted up to 3 min, and the time during which the rat was able to stay on the roller was recorded [30]. Three trials were performed, and the average value was calculated.

To eliminate bias, MEPs and balance and postural orientation tests were performed by an experienced physiatrist blinded to the group allocations.

### Statistical analysis

When the Kolmogorov–Smirnov test and Shapiro–Wilk test were performed to use the parametric method, the normality was not satisfied. The Mann–Whitney U test was used to compare the values of electrophysiological measurements and balance and postural orientation tests between the tDCS and control groups. The Wilcoxon signed rank test was used to compare the values of electrophysiological measurements between day 3 and day 4, and to compare the values of the balance and postural orientation tests between day 3 and day 5 in the tDCS and control groups, respectively. Statistical analysis was performed using SPSS version 21.0 (IBM SPSS, Armonk, NY, USA), and p-values less than 0.05, were considered statistically significant.

## Results

### MEPs

**Fig. 5:**
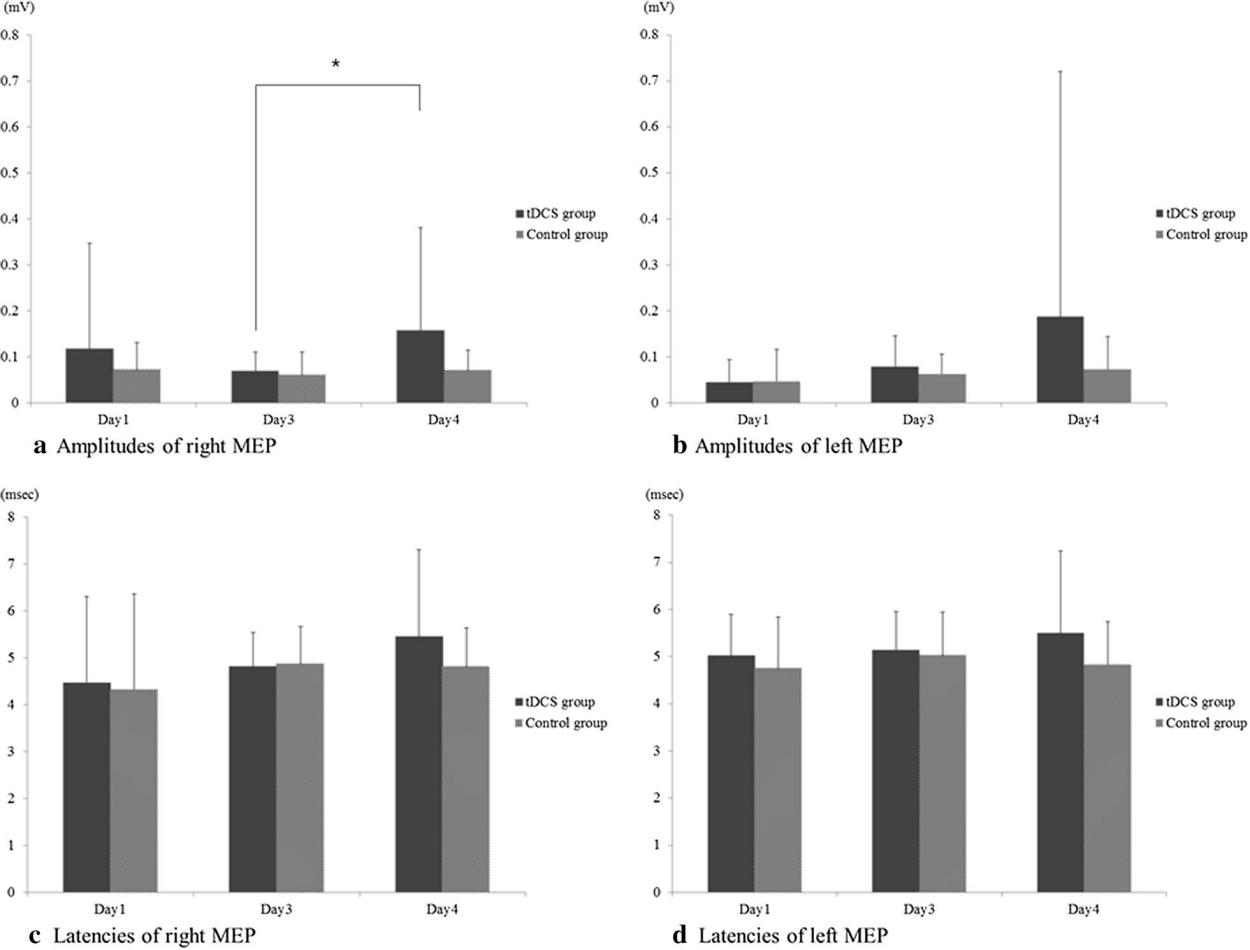
Results of MEP study. **b** In the tDCS group, the right MEP amplitudes are significantly different between days 3 and 4. **b** No significant difference is found between the left MEP amplitudes. **c**, **d** No significant difference is found between the bilateral MEP latencies

The amplitudes and latencies of MEP on days 1, 3, and 4 showed no significant difference between the tDCS group and the control group. The amplitude of the right MEP recorded from the tibialis anterior muscle of the right hindlimb, which resulted from left motor cortex stimulation, progressively declined from day 1 to 3 in both groups. The amplitude of right MEP was significantly different between day 3 and day 4 in the tDCS group (0.069 ± 0.042 versus 0.158 ± 0.223 mV, p = 0.041) (Table 1; Fig. 5a), whereas there was no significant difference between day 3 and day 4 in the control group. There was no significant difference in the amplitudes of the right MEP between the tDCS group and the control group measured on day 4 (Fig. 5a). In addition, no significant difference was observed in the bilateral MEP latencies recorded at the bilateral tibialis anterior muscles between days 3 and 4 (Fig. 5c, d).

**Table 1:** The results of motor-evoked potential (MEP) evaluation performed in each group

Evaluation	tDCS group			Control group		
	Day 1	Day 3	Day 4	Day 1	Day 3	Day 4
Right MEP^†^ amplitude (mV)	0.117 ± 0.230	0.069 ± 0.042	0.158 ± 0.223*	0.072 ± 0.059	0.060 ± 0.051	0.070 ± 0.045
Left MEP^††^ amplitude (mV)	0.045 ± 0.049	0.079 ± 0.067	0.187 ± 0.533	0.046 ± 0.070	0.063 ± 0.043	0.072 ± 0.072
Right MEP^†^ latency (ms)	4.471 ± 1.830	4.813 ± 0.721	5.451 ± 1.850	4.313 ± 2.055	4.873 ± 0.800	4.815 ± 0.821
Left MEP^††^ latency (ms)	5.022 ± 0.870	5.137 ± 0.825	5.497 ± 1.745	4.751 ± 1.088	5.026 ± 0.923	4.832 ± 0.904

Values are mean ± standard deviation

^†^Right MEP, MEP recorded at right hind limb

^††^Left MEP, MEP recorded at the left hind limb

^*^p < 0.05

In this study, since tDCS was applied to the left M1 area, the measurement of the change in the right MEP was of interest, and the left MEP was used as a control for the MEP test. There was no significant difference in the amplitudes of the left MEP recorded at the left tibialis anterior muscle between days 3 and 4 in each group (Fig. 5b). There was no significant difference in the amplitudes of the left MEP between the tDCS and control groups measured on day 4 (Fig. 5b).

### Balance and postural orientation tests

**Fig. 6:**
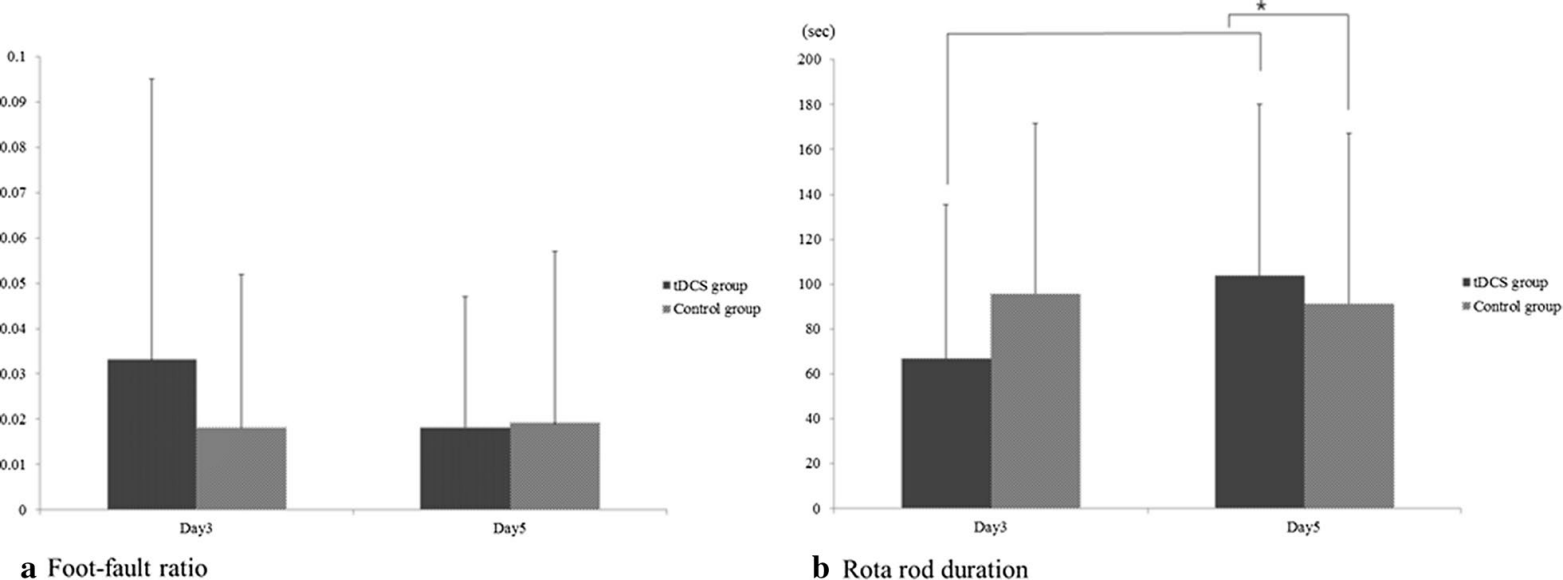
Results of behavioral studies. **a** The foot-fault ratio is slightly lower in the tDCS group than in the control group, however, the results are not statistically significant. **b** The rotarod duration on day 5 is significantly increased as compared with that on day 3 in the tDCS group. **c** The rotarod durations on day 5 are significantly different between the tDCS group and the control group

Although not statistically significant, the foot fault ratio showed declining trend when comparing day 3 and day 5 in the tDCS group (Table 2; Fig. 6a).

**Table 2:** The results of motor coordination studies performed in each group

Evaluation	tDCS group			Control group		
	Day 1 (Pre- repetitive mTBI)	Day 3 (Pre-tDCS)	Day 5 (Post-tDCS)	Day 1 (Pre- repetitive mTBI)	Day 3 (Pre-tDCS)	Day 5 (Post-tDCS)
Foot-fault ratio	0041 ± 0.028	0.033 ± 0.062	0.015 ± 0.029	0.032 ± 0.035	0.018 ± 0.034	0.019 ± 0.038
Rota rod duration (sec)	40.561 ± 47.346	66.621 ± 68.797	103.697 ± 76.470*	33.995 ± 44.437	95.422 ± 76.166	91.141 ± 76.146

Values are mean ± standard deviation

*mTBI* mild traumatic brain injury, *tDCS* transcranial direct current stimulation

^*^p < 0.05

Based on the results of the foot-fault test, the foot-fault ratio in the tDCS group was slightly lower than that of the control group, however, this difference was not statistically significant (Table 2; Fig. 6a).

The rotarod duration was significantly increased on day 5 compared with day 3 in the tDCS group (66.621 ± 68.797 s versus 103.697 ± 76.470 s, p = 0.001) (Fig. 6b). There was a significant difference between the rotarod durations of the tDCS group and the control group measured on day 5 (103.697 ± 76.470 versus 91.141 ± 76.146 s, p = 0.006).

In the control group, there was no statistically significant difference in the results of the foot-fault test and rotarod test between days 3 and 5.

## Discussion

n this study, the influence of anodal tDCS at the M1 area on amplitude of MEP and balance function after repetitive mTBI was evaluated. The results demonstrated that anodal tDCS at the M1 area, which underwent immediately after repetitive mTBI, increased the amplitude of MEP, showed a decreasing trend of the foot-fault ratio, and improved the rotarod duration. tDCS may have therapeutic benefits for balance function and electrophysiological changes. To the best of our knowledge, this study is the first to suggest an increase in corticospinal excitability and balance improvement after anodal tDCS on the M1 area in a large number of repetitive mTBI rat models by analyzing MEP studies and behavioral tests.

Standing and gait are critical for most activities of daily living. In patients with mTBI, balance instability and gait alteration are predictors of increased fall risk, loss of functional independence, morbidity, and mortality. A previous study showed that mTBI patients had a greater sway area, larger mediolateral displacement amplitude, and slower body oscillation than healthy individuals [31]. Martini et al. showed that mTBI patients adopted a more conservative gait strategy and slower gait than those without a history of mTBI [32, 33]. To maintain balance, the central nervous system must effectively integrate sensory and motor information through complex mechanisms involving cortical and subcortical pathways [34]. Previous studies suggest that combinations of central and peripheral deficits may be the cause of balance and gait alterations in mTBI [35, 36]. Balance and gait alteration occurs through the following two mechanisms: (1) the brain centers responsible for central integration of vestibular, visual, and somatosensory information may be impaired, or (2) the peripheral receptors themselves may be damaged and provide inaccurate senses of motion. A recent study showed that a higher incidence of asymmetry of the corticospinal tract (CST) was found in patients with mTBI [37]. The injury to the CST leads to problems in balance and coordination [37]. Furthermore, Karayannidou et al.'s study provided insight into how pyramidal tract neurons (PTNs) from the fore- and hindlimb projections in the primary motor cortex respond to postural changes during two distinct tasks [38]. PTNs are the main output neurons of the motor cortex and influence the activity of motor neurons and interneurons in the ventral horn of the spinal cord. In other words, it may be assumed that anodal tDCS on the M1 area would have improved balance by activating the CST as well as the PTNs.

tDCS is a promising strategy to modulate brain network function and, in doing so, the supraspinal control of balance. tDCS safely and selectively modulates the excitability of brain networks [39]. tDCS targeting the primary sensorimotor regions has been demonstrated to improve balance in older adults and patients with stroke [40–42]. The M1 area is composed of the primary motor cortex and premotor cortex [43]. The CST originates from several cortical areas, and approximately half of these axons extend from neurons in the primary motor cortex. The primary function of the CST is voluntary motor control of the body and limbs. In this study, the amplitudes of the right MEP progressively declined from day 1 to 3 in both groups. This result demonstrated the disruption of the CST by repeated mTBI. This would have resulted in balance and gait alterations in the repetitive mTBI rat model. Anodal tDCS to the M1 area of repetitive mTBI rat models generates an electric field that polarizes neuronal populations and modulates resting membrane potentials. This activates the CST and induces balance and gait improvements.

In this study, a progressive decrease in the amplitude of right MEP after repetitive mTBI compared with before mTBI, and a significant increase in the amplitude of right MEP after tDCS treatment compared with before tDCS treatment, was observed. The decreased MEP amplitude is evidence of decreased cortical excitability following repetitive mTBI, and the increased MEP amplitude is evidence of increased corticospinal excitability following anodal tDCS [44]. MEP amplitude represents the total number of depolarized axons and innervated muscle fibers, and their degree of excitability [44]. Therefore, our results suggest that the number of motor units involved in corticospinal excitability was reduced by repetitive mTBI and could be restored by anodal tDCS in the M1 area. Replacement of lost fibers by the central nervous system may be facilitated by simultaneous excitement of motor units [45]. In contrast, no significant change was observed in the MEP latency of either group. The MEP latency reflects the speed at which signals arrive through the fastest-conducting nerve fibers. This suggests a mechanism in which anodal tDCS increases the number of axons involved in excitation but does not increase conduction velocity. These results are consistent with our previous study; the amplitude of MEP decreased after repetitive mTBI and increased after tDCS, but MEP latency was not affected [20].

In a previous tDCS study of the repetitive mTBI rat model, the sensory-evoked potential and MEP were measured to confirm transient motor recovery [20]. A previous study did not focus on balance, motor coordination, or gait impairment, and no behavioral tests were performed [20]. In addition, a previous study showed that a single session of anodal tDCS had little effect on balance improvement and postural control [46]. However, in this study, it was found that single-session tDCS helped in the activation of the CST and improvement of balance. Another study suggested that multi-session anodal tDCS has an effect on balance improvement in children with cerebral palsy [47, 48]. In order to further confirm the results of this study, it would be necessary to conduct comparative studies on multi-session anodal tDCS and single-session anodal tDCS in a repetitive mTBI rat model in the future.

Balance and posture are controlled by numerous interacting networks, such as the spinal cord, cerebellum, cortex, and brainstem [49, 50]. Yosephi et al.’s have suggested that bilateral stimulation of the cerebellar hemispheres is more effective than M1 area stimulation when anodal tDCS is implemented for the purpose of improving balance in older adults with high risk of falls [51]. However, a previous study showed that mTBI is associated with white matter and gray matter volume reduction and cortical thinning in areas including the M1 area, but does not affect the cerebellum [52]. Therefore, in this study, to confirm the effect of tDCS on balance improvement, the M1 area was stimulated rather than the cerebellum.

The rat is a key model for basic and preclinical studies of neuroscience, underlining its importance in studies of human disease. There are the close evolutionary and genomic relationship to humans, the sophistication and sociability of the animal, and the ease of physiological and behavioral measurements. Anodal tDCS on the M1 for balance and gait in rat model could be useful to further explore insight and may serve as a translational platform bridging human and animal studies, establishing new therapeutic strategies for repetitive mTBI.

### Limitation

The present study has several limitations. First, a single session of anodal tDCS may be insufficient to significantly improve motor coordination and electrophysiology. Multiple sessions of anodal tDCS may produce clearer results than the current results; therefore, further research with multiple sessions of anodal tDCS in a repetitive mTBI rat model is needed. Second, in the present study, brain MRI was conducted 4 days after the first induction of mTBI and 2 days after the last induction of mTBI. It is known that repetitive mTBI can cause structural changes in the brain, such as cortical thinning and ventriculomegaly. In this study, brain MRI was conducted to evaluate the possibility that the weight drop device produced a skull fracture or brain hemorrhage. If brain MRI was performed after a longer period of time, changes in brain structure due to repetitive mTBI could have been confirmed in addition to ruling out skull fracture or hemorrhage. Third, as the rates of mTBI are higher in females than in males when similar sports are compared, anodal tDCS study on female are also needed [18]. In future study, the study on the effect of anodal tDCS at the M1 area in repetitive mTBI considering female's menstrual cycle would be needed. Fourth, immunochemical analysis was not conducted in this study. In subsequent studies, it would be necessary to apply some immunohistological analysis; antibody against BDNF to investigate effects on neural plasticity, GFAB to confirm the reactive astrocytosis and vGlut1 and GAD 65–67 markers to assess glutamate and GABA levels [24, 53, 54]. And in this study, the number of anesthesia was minimized to reduce side effects caused by anesthesia [55, 56]. For this reason, MEP after tDCS was conducted on day 4 and motor coordination studies were performed on day 5. There is sufficient time interval for neural plasticity to occur, and it makes some difficulty on the correlation between MEP and changes in behavioral performance. Since the MEP and motor coordination studies were examined on different days, it is difficult to prove that the results of the motor coordination study were not due to motor learning but due to an increase in corticospinal excitability. In the follow-up study, the correlation between the two tests would be increased by reducing the temporal interval between the two tests.

## Conclusions

Balance impairments can increase the risk of falling, which may lead to the loss of functional independence and severe injuries. Therefore, improvement of balance and motor coordination function is an important treatment consideration in patients with repetitive mTBI to reduce the risk of falls and its consequences. This study proposes that anodal tDCS at the M1 area after repetitive mTBI could improve the amplitude of MEP as well as improve balance control, postural orientation and motor endurance by activating the CST. These results indicate that anodal tDCS can produce rapid, consistent, and controllable electrophysiological changes in corticomotor excitability in repetitive mTBI rat models. This newly developed tDCS protocol in a repetitive mTBI rat model may serve as a translational platform bridging human and animal studies, establishing new therapeutic strategies for patients with repetitive mTBI.

## Data Availability

Data will be available upon reasonable request to the corresponding author.

## Abbreviations

mTBI: Mild traumatic brain injury
M1: Primary motor cortex
tDCS: Transcranial direct current stimulation
MEP: Motor-evoked potential
MO: Machine output
CST: Corticospinal tract
PTN: Pyramidal tract neuron
